# Age-associated changes in innate and adaptive immunity: role of the gut microbiota

**DOI:** 10.3389/fimmu.2024.1421062

**Published:** 2024-09-16

**Authors:** Haoyu Gao, Eugenie Nepovimova, Vojtech Adam, Zbynek Heger, Marian Valko, Qinghua Wu, Kamil Kuca

**Affiliations:** ^1^ College of Life Science, Yangtze University, Jingzhou, China; ^2^ Department of Chemistry, Faculty of Science, University of Hradec Králové, Hradec Králové, Czechia; ^3^ Department of Chemistry and Biochemistry, Mendel University in Brno, Brno, Czechia; ^4^ Faculty of Chemical and Food Technology, Slovak University of Technology, Bratislava, Slovakia; ^5^ Andalusian Research Institute in Data Science and Computational Intelligence (DaSCI), University of Granada, Granada, Spain

**Keywords:** aging, innate immunity, adaptive immunity, cGAS-STING, gut microbiota aging, gut microbiota

## Abstract

Aging is generally regarded as an irreversible process, and its intricate relationship with the immune system has garnered significant attention due to its profound implications for the health and well-being of the aging population. As people age, a multitude of alterations occur within the immune system, affecting both innate and adaptive immunity. In the realm of innate immunity, aging brings about changes in the number and function of various immune cells, including neutrophils, monocytes, and macrophages. Additionally, certain immune pathways, like the cGAS-STING, become activated. These alterations can potentially result in telomere damage, the disruption of cytokine signaling, and impaired recognition of pathogens. The adaptive immune system, too, undergoes a myriad of changes as age advances. These include shifts in the number, frequency, subtype, and function of T cells and B cells. Furthermore, the human gut microbiota undergoes dynamic changes as a part of the aging process. Notably, the interplay between immune changes and gut microbiota highlights the gut’s role in modulating immune responses and maintaining immune homeostasis. The gut microbiota of centenarians exhibits characteristics akin to those found in young individuals, setting it apart from the microbiota observed in typical elderly individuals. This review delves into the current understanding of how aging impacts the immune system and suggests potential strategies for reversing aging through interventions in immune factors.

## 1 Introduction

Ever since Roy Walford first proposed the idea in 1962 that aging might be intricately linked to the immune system’s histoincompatibility response (1), the connection between aging and immune functionality has remained a prominent focus within gerontological research. As individuals age, their biological systems undergo an irreversible decline in normal physiological functions, such as memory deterioration (2), reduced secretion of pertinent hormones (3), degenerative changes in tissues and organs (4), and a weakened immune defense (5).

Aging is accompanied by a compromised immune response, while the bone marrow, thymus, and secondary lymphoid organs exhibit degeneration in the elderly when compared to the young. Within the bone marrow, hematopoietic stem cells can differentiate into myeloid cells (such as granulocytes, monocytes, and dendritic cells) as well as lymphoid cells (such as T cells, B cells, and natural killer (NK) cells) (6, 7). However, with age, the density of human bone marrow and angiogenesis decrease (8), which are coupled with a reduction of hematopoietic stem cell regeneration potential and the alteration of its epigenetic markers (9, 10). These changes might contribute to age-related immunodeficiency. Thymus serves as the primary site for T cell development (11), and the precisely organized structure of secondary lymphoid organs plays a crucial role in establishing distinct T and B cell compartments (12). The involution of these organs with advancing age may have a negative impact on the spatial and temporal interactions between stromal cells and lymphocytes, thus can jeopardize the survival of naïve T cells and impede the effective elimination of autoreactive lymphocytes (13–15). These age-related immune system changes, including both the innate and adaptive branches, are primarily manifested as a diminished immune response to exogenous and endogenous antigens, a reduced capacity to react to novel antigens, delayed responsiveness to the protective effects of vaccines, and weakened immune memory (16, 17). Consequently, the overall ability of the individual to fend off infectious diseases, combat tumors, and clear out senescent cells wanes with age (18). This age-related impairment in immune function is coined as “immunosenescence.”

Immunosenescence encompasses a comprehensive array of age-related alterations within the immune system, including both innate and adaptive immunity, along with imbalances between these two components (19). Notable examples include shifts in the quantity and functionality of neutrophils (20), monocytes (21), macrophages (22), NK cells (23), mast cells (24), and related cytokines in innate immunity; alteration in the number and function of T cells (25) and B cells (26) in adaptive immunity. Current studies have highlighted a more pronounced influence of aging on the adaptive immune system compared to the innate counterpart (27), and the impairment of immune surveillance will accelerate the accumulation of senescent cells and further expedite the aging process (28). However, whether in the context of health or disease, aging and immunosenescence are closely related but not equivalent concepts. Most aging individuals experience immunosenescence, but the degree of immune function decline varies greatly from individual to individual. At the same time, certain diseases may induce immune system changes resembling those seen in immunosenescence even in young patients (29). Therefore, the intricate interplay between “aging” and “immunity” necessitates nuanced analysis considering different categories, properties, and immune backgrounds. Most of the research on the immune system and organismal aging has been established at the level of immune cells, but the exploration of the cGAS-STING pathway illuminates the mechanisms through which DNA triggers innate immunity. In addition, investigations into alterations in gut microbiota during aging and the potential for gut microbiota rejuvenation in long-lived elderly individuals offer promising avenues to unravel the complex relationship between immunity and aging.

This review explores the effects of aging on both the innate and adaptive immune systems. We delve into the dynamic changes observed in the gut microbiota as humans age, highlighting the distinct gut microbiota found in long-lived elderly individuals. Furthermore, the review addresses contemporary clinical approaches to anti-aging, specifically focusing on strategies aimed at prolonging healthy lifespan by rejuvenating the immune system in the elderly.

## 2 Aging and the innate immune system

**Figure 1:**
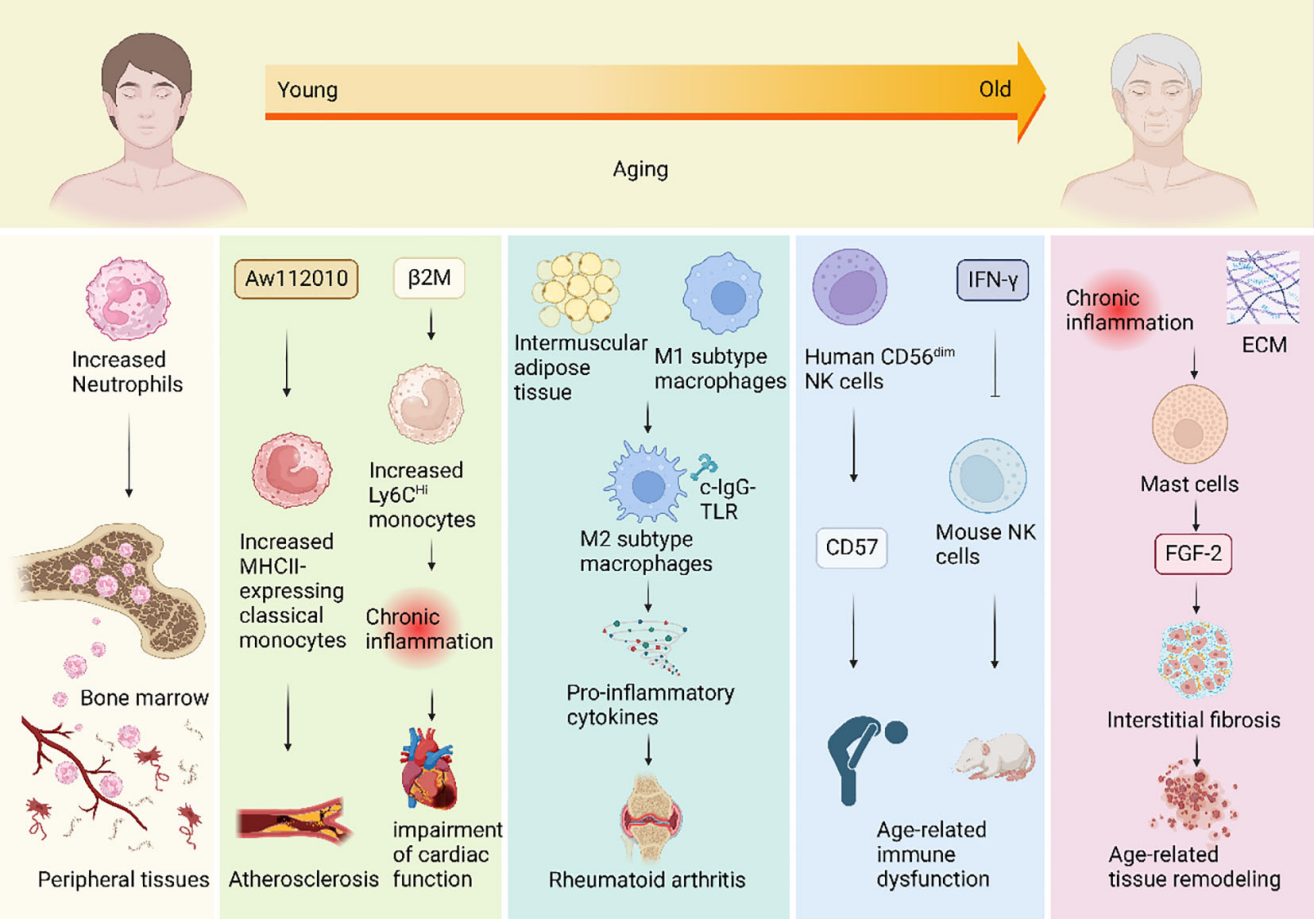
Mechanisms of changes in innate immunity during human aging. During the aging process, the innate immune system undergoes changes that impact various immune cells, giving rise to a spectrum of aging-related diseases. Neutrophil counts increase with age and they migrate from the bone marrow to infiltrate peripheral tissues. Additionally, an upregulation of the inflammatory regulator gene *Aw112010* results in an elevated number of classical monocytes expressing the gene MHCII in aging mice, potentially triggering atherosclerosis. The aging-related rise in beta-2 microglobulin (β2M) levels is positively correlated with age and may contribute to an increase in circulating Ly6C^Hi^ monocytes. This elevation could lead to chronic inflammation, adversely affecting cardiac function. Moreover, aging induces the polarization of macrophages from the M1 subtype to the M2 subtype by expanding intermuscular adipose tissue in skeletal muscles. When subjected to complexed immunoglobulin G Toll-like receptor (c-IgG-TLR) stimulation, anti-inflammatory M2 macrophages can paradoxically produce pro-inflammatory cytokines, potentially contributing to conditions like rheumatoid arthritis. In the elderly, the number of CD56^dim^ NK cells significantly increases, accompanied by heightened expression of the inhibitory receptor CD57, which may underlie age-related immune dysfunction. Furthermore, chronic inflammation in old age, coupled with increased extracellular matrix (ECM) levels, may contribute to elevated mast cell numbers. Concomitant with the rise in mast cell numbers, the expression level of FDF-2 in rats also increases, suggesting mast cells’ potential involvement in age-related tissue remodeling by promoting fibrosis.

Aging is an irreversible natural development process of organisms. Consisting of different cells and factors, innate immunity (also known as nonspecific immunity or congenital immunity) is the first line of defense against infection (30, 31). Organisms directly resist the invasion of microorganisms to the host by identifying pathogens. Many aspects of the effector functions of innate immune cells, including neutrophils, monocytes, macrophages, natural killer cells, mast cells, and dendritic cells, change with age (32) (**Figure 1**).

### 2.1 Aging and neutrophils

Formed from stem cells in the bone marrow (33, 34), neutrophils, the most abundant types of granulocytes in human leukocyte population, are an important part of the innate immune system (34–36). When infection occurs, neutrophils are the first to reach the inflammatory site to kill pathogens through phagocytosis and degranulation (37–39). In addition, neutrophils recruit other immune cells by releasing cytokines and chemokines and exert an important function in coordinating innate and adaptive immune responses through antigen presentation (40, 41). Therefore, neutrophils play an important role in inflammation. However, the relationship between neutrophils and aging is presently not very clear. In the process of aging, neutrophils will migrate from bone marrow and infiltrate into peripheral tissues, such as white and brown adipose tissue, liver tissue, and so on (42). Previous studies have suggested that the number of neutrophils remains stable in healthy elderly (43–45). Yet, recent studies have found that the number of neutrophils in the elderly increases relatively with age and shows dysfunctional phagocytosis and chemotaxis (46–48), which may be linked to the decrease of apoptosis caused by neutrophils activated by chronic inflammation (49). CD11b and HLA-DR are markers indicating neutrophil activation. In experiments using CD11b^++^ and HLA-DR^+^ neutrophils, it was found that the increase of age can also lead to the upregulation of inflammation-related circulating products tumor necrosis factor (TNF) and circulating mitochondrial DNA (mtDNA) (50), which may also be a major reason for the increase in neutrophils with age (48). Compared with the young, the proliferation of neutrophil precursors in the bone marrow of the elderly is significantly reduced, so the decrease in neutrophils is considered to be the main reason for the increase in infectious events in the elderly (51). Aging is also a factor leading to the decrease of neutrophils, which is often associated with neutropenia (52–55). Previous studies have suggested that ≥65 years old is one of the triggers for neutropenia (54, 56–58). Other studies have found that there is a significant correlation between age and the incidence of febrile neutropenia in patients with small cell carcinoma treated with cisplatin plus etoposide and carboplatin plus etoposide (52).

Some studies suggested that neutrophils may induce senescence by causing telomere damage *in vitro and ex vivo*, which is ROS-dependent and may be related to organismal aging. For example, when neutrophils were co-cultured with fibroblasts, the ROS released by the neutrophils significantly reduced the telomere FISH intensity in the fibroblasts and markedly increased their telomere-associated foci, thereby inducing senescence. However, these effects could be prevented by adding catalase (59). In addition, the number of hepatocytes with telomere dysfunction increased with age in aged mice (60). Exposing precision-cut liver slices (PCLS) to wild-type neutrophils revealed increased expression of the senescence markers p21^CIP1^ and p16^INK4A^ in PCLS; however, this effect was abolished when neutrophils from transgenic mice overexpressing human catalase in mitochondria were added (59).

Overall, these findings shed light on the complex dynamics that govern the connection between age and neutrophils, highlighting the multifaceted nature of this relationship (**Table 1**). The numbers of neutrophils can either decrease, increase, or remain stable with age, depending on a range of influential factors. These factors include chronic inflammation, upregulation of TNF and mtDNA, increased risk of comorbidities, race, low-performance status, male gender, and previous radiotherapy. The interplay of these elements collectively shapes the impact of age on neutrophil levels. In addition, neutrophils may play a role in triggering organismal aging, primarily through an indirect pathway involving the induction of cellular senescence. This process is initiated by oxidative damage, which in turn leads to telomere dysfunction. Therefore, although neutrophils are recognized for their pivotal role in microbial killing and inflammation, their involvement in the aging process and potential long-term negative effects on the body should not be overlooked.

**Table 1:** Changes in innate immune system during aging.

Immune cells	Models	Expression changes	Mechanisms	References
**CD11b^++^ neutrophils**	The advanced-age, frail elderly	Increase	TNF is upregulated in the serum of frail elderly donors	(48)
**HLA-DR^+^ neutrophils**	The advanced-age, frail elderly	Increase	Exogenous mtDNA can stimulate blood neutrophils to express HLA-DR in a dose-dependent manner	(48)
**CD14^high^CD16^−^/CD14^high^CD16^+^/CD14^low^CD16^+^ classical/intermediate/non-classical monocytes**	Human peripheral blood mononuclear cells	Classical monocytes: numbers increase, but frequencies decrease Intermediate/Non-classical monocytes: frequencies and numbers increase	Monocytes display increased immune activation marker levels, decreased co-inhibitory molecule expression, elevated CCR2 expression on classical monocytes, and reduced CX3CR1 expression on non-classical monocytes.	(63)
**Ly6C^hi^/Ly6C^int^/Ly6C^lo^ classical/intermediate/non-classical monocytes**	Blood/spleen/bone marrow of mice	Classical monocytes: increase in the blood and spleen Intermediate/Non-classical monocytes: increase	Aging leads to an augmentation in both classical and non-classical monocyte counts, which may be attributed to myeloid-biased hematopoiesis. Elevations in the pro-aging factor plasma β2 microglobulin are responsible for the rise in circulating Ly6C^Hi^ monocytes.	(21, 66)
**M1 macrophages**	Rat hepatic macrophages	CD68^+^ macrophages increase	Excessive iron levels and age-related conditions.	(73)
**M1/M2 macrophages**	Human skeletal muscle	CD68^+^/CD80^+^ macrophages decrease whereas CD68^+^/CD206^+^ macrophages increase	Progressive accumulation of intermuscular adipose tissue (IMAT) in aging skeletal muscle.	(70)
**M2 macrophages**	Rat hepatic macrophages	CD163^+^ macrophages increase	Excessive iron levels and age-related conditions	(73)
**F4/80^+^ CD11b^+^ macrophages**	Male mouse peritoneal macrophages	Frequencies decrease	The functionality of macrophages is compromised by both aging and protein malnutrition.	(77)
**CD56^dim^ NK cells**	Human peripheral blood	Increase	The rise in the absolute count of CD56^dim^ cells could indicate a compensatory mechanism in response to alterations in receptor expression.	(85)
**CD56^dim^/CD56^bright^ NK cells**	Human peripheral blood	Stable/decrease	Impaired immune regulation in the elderly is associated with a decline in CD56^bright^ NK cells.	(84)
**CD56^bright^ NK cells**	Human peripheral blood	Frequencies decrease, but numbers are stable	CD56^bright^ cells represent immature NK cells that have the capacity to undergo differentiation into CD56^dim^ cells both *in vitro* and *in vivo*.	(85, 86)
**NKG2C^+^ CD122^low^ NK2 cells**	Human peripheral blood	Increase	The NK2 subpopulation represents a phenotypically memory-like subset of NK cells, and it is notably linked to the aging process.	(91)
**Tryptase^+/-^/chymase^+/-^ mast cells**	Human epithelial tissues	Frequencies remain stable	The quantity of mast cells rises, yet the frequency of cells identified through dual labeling remains constant in aged skin.	(122)

### 2.2 Aging, monocytes, and macrophages

Aging is a complex process that involves multiple biological and biochemical mechanisms. Monocytes and macrophages, two key cell types in the innate immune system, also play important roles in the aging process. Both monocytes and macrophages belong to phagocytes. Monocytes are generally considered to be the precursors of macrophages, but current studies suggest that most macrophages are embryonic-derived (61, 62). With the aging of the organism, the phenotype and function of monocytes and macrophages in the elderly may change.

As the body ages, monocytes will change. In both aged mice and humans, the total number of circulating monocytes increases with age across classical, intermediate, and non-classical subsets (21, 63), while the percentage of classical monocytes relative to the total monocyte population decreases in the elderly (63). Plasma beta-2 microglobulin (β2M) is mainly derived from platelets (64) and positively correlated with age (65). An increase in β2M partially leads to an increase in circulating Ly6C^Hi^ monocytes. When platelet-specific *β2M* was knocked out (Plt-β2M^-/-^), aged mice had significantly fewer circulating Ly6C^Hi^ monocytes but more pro-reparative genes *Il10, Il27*, and *Cxcl12*, exhibiting an anti-inflammatory phenotype (66). This seems to suggest that aging can be delayed by reducing Ly6C^Hi^ pro-inflammatory monocytes. However, aged mice exhibited more aging phenotypes, such as reduced heart function (64). Inflammation, while harmful in some cases, can also help maintain heart function in others (67). Therefore, it seems that people should center more attention on maintaining immune homeostasis than preventing chronic inflammation. Furthermore, the number of classical monocytes with MHCII genes increased during aging in mice, which may be due to the increased expression of inflammatory regulator *Aw112010* (21). In addition to quantitative changes, aging can also lead to changes in the function of monocytes. Aged monocytes are known to have reduced cellular effector capacity. The main manifestation is the impaired phagocytosis of monocytes, which leads to telomere shortening under the stimulation of Toll-like receptor (TLR) 4, significantly increased levels of intracellular tumor necrosis factor (TNF)-α level (63, 68), and decreased production of interleukin (IL)-1 (69) and IL-6 (63). Further, aging also decreased the expression of co-inhibitory molecules 2B4 (CD244), T-cell immunoglobulin domain and mucin domain 3 (TIM-3), CD200R, T-cell immunoglobulin and ITIM domain (TIGIT), and B and T lymphocyte attenuator (BTLA), while increased the expression of immune activation markers human leukocyte antigen-DR (HLA-DR), cluster of differentiation molecule 11b (CD11b), and L-selectin (CD62L) (63).

Compared with monocytes, much more effort has been devoted to studying the effects of aging on macrophages. The total number of macrophages remains stable in aged skeletal muscle, most of which are the anti-inflammatory M2 subtype, proportional to age, and a small part are M1 subtypes, which are inversely proportional to age (70). This seems to contradict the pro-inflammatory state of aging. In fact, M2 macrophages can also produce pro-inflammatory cytokines in response to complexed immunoglobulin G (c-IgG)-TLR stimulation (71), and macrophages are not the only cells that cause systemic inflammation due to aging (72). The reason why M2 becomes the main subtype of macrophages in skeletal muscle in the elderly may be intertwined with the increase of intermuscular adipose tissue in aged skeletal muscle (70). Aging can also lead to hepatic iron deposition and the accumulation of M1 and M2 hepatic macrophages, but no changes in the phenotype and number of macrophages were observed by administering iron dextran to mice, signifying that iron may not be the cause of the increase in liver macrophages due to aging (73). Paradoxically, iron can both promote the production of pro-inflammatory cytokines such as IL-6 and TNF-α, thereby increasing the number of macrophages (74), and accumulate continuously with age in monocytes, thus differentiating into macrophages (75). The reason for this contradictory phenomenon may lie in the different types of cellular iron. Malnutrition is one of the predisposing problems in the elderly population (76), and studies have linked macrophages to the disease. Compared with young malnourished animals, the number of F4/80^+^ CD11b^+^ macrophages was significantly decreased in aged malnourished animals, but there was no significant difference compared with aged control animals (77). CD86, one of the most representative surface markers of M1 macrophages (78), was also found to be reduced in aged animals, but it was not associated with malnutrition (77), which partly confirms that macrophages in aged animals are polarized to M2 subtype. TLR-4, the receptor of lipopolysaccharide (LPS) (79), plays a critical role in regulating the innate immune system (80). The expression of this receptor is decreased in the malnourished population, but this decrease is not implicated in age (81). Through this experiment, we can find that aging may lead to the decrease of M1 macrophages, but this decrease has nothing to do with malnutrition. Malnutrition may lead to a decrease in TLR-4 expression, but this decrease has nothing to do with aging (81). Therefore, based on the above findings, we conclude that aging or malnutrition induces the polarization of macrophages from pro-inflammatory M1 to anti-inflammatory M2 subtypes, adversely affecting the immune response. However, it is impossible to determine from this review if there are any interactions between aging and malnutrition. Albumin, a biological indicator of malnutrition, has been found to decrease with age in the elderly population (82), which directly links aging with malnutrition. Future research can utilize albumin as a medium to explore the connections among aging, malnutrition, and macrophages, thereby exploring the immune mechanism in greater depth.

In summary, the number of monocytes tends to increase with age. However, the knockout of platelet-specific *β2M* can inhibit the pro-inflammatory differentiation of monocytes in aged mice, suggesting that inflammation may not be entirely harmful to the human body. In fact, acute inflammation might be beneficial in some ways. Acute inflammation is the body’s response to infection and injury, aiding in the healing process. For example, when a person is injured or contracts a virus such as a cold or flu, the immune system releases white blood cells to the affected area. These cells surround and protect the injured site, playing a crucial role in the body’s healing mechanism. Nevertheless, aging can still lead to monocyte dysfunction and shortened telomere length, affecting their functionality. Regarding macrophages, their numbers typically remain stable in skeletal muscle as individuals age. The accumulation of hepatic macrophages in aging may not be directly linked to iron deposition, but previous studies have shown that iron can indirectly promote the increase of macrophages by generating pro-inflammatory cytokines such as IL-6 and TNF-α and accumulating in monocytes. However, the impact of long-term changes in iron metabolism on macrophages requires further investigation. Moreover, the high expression of IL-6 and TNF-α can also lead to a decrease in albumin (83), a biological indicator of malnutrition in elderly individuals. This finding might explain why the elderly, considered a high-risk group for malnutrition, experience reduced M1 macrophages after a high-fat diet. In conclusion, we shed light on the complex relationships between monocytes, macrophages, and aging (**Table 1**). Further research is necessary to fully understand these interactions and their implications for human health.

### 2.3 Aging and natural killer cells

NK cells are a group of toxic lymphocytes defined by CD3^-^, CD16^+^, and CD56^+^ phenotypes, which belong to a type of lymphocytes and can directly kill virus-infected cells, tumor cells, and abnormal cells. With age, the frequency and composition of NK cells may change. Previous studies found that the count of NK cells remains constant with age. For instance, the number of CD56^dim^ NK cells was independent of aging (84). Recent studies have found that the frequency and absolute number of total NK cells and CD56^dim^ NK cell subset were stable in the elderly compared to adults. In contrast, the frequency of CD56^bright^ NK cell subsets was strikingly reduced in the elderly, although the absolute number of CD56^bright^ NK cell subsets were stable when compared with adults (85, 86). The two experiments mentioned above have reached different conclusions regarding “the impact of aging on the absolute number and frequency of NK cells”. This discrepancy may be due to several factors: the different definitions of the age span of adults (the former is 20-40 years old; the latter is 19-59 years old), the distinct health status of the subjects and the limitations of techniques. For mice, since CD56 is not expressed, subset classification cannot be performed based on CD56, but can be divided according to CD27 (87). Aging leads to significant changes in the distribution of NK cells in different tissues of mice. Aged mice show a significant decrease in the frequency and number of NK cells in their blood, spleen, and liver compared to young mice (88), indicative of a decline in the immune function of aged mice, as NK cells play an important role in defending against viral infections and cancer. Although the proportion of NK cells in the immune system of the lung is altered in aged mice, the total number of NK cells in the lung remains stable (88). Moreover, the frequency and number of NK cells in the lymph nodes of aged mice tend to increase, albeit not significantly (88). This may suggest that the immune system of aged mice is compensating for the decline in NK cells in other organs by increasing the number of NK cells in lymph nodes. Memory-like NK cells (NK2, NKG2C^+^ CD122^low^) exhibit distinct characteristics, including lower expression of FcϵRγ (*FCER1G*), SYK (*SYK*), EAT-2 (*SH2D1B*), PLZF (*ZBTB16*) and higher NKG2C (*KLRC2*) compared with other NK cell subpopulations (89, 90). These cells have garnered significant attention for their robust response to viruses and their tendency to accumulate with age (91). However, the development process, cell life of memory-like NK cells and which subset of mature NK cells they are transformed from await further studies to be clarified.

In addition to the effect on the frequency of NK cells and circulating NK cell pool, aging also has an impact on NK cell phenotype and function. CD16, also known as FcγRIII, is an Fc receptor that can bind antibodies and virus particles to activate the killing effect of NK cells. It was found that the expression of CD16 in elderly donors has a slight but not significant extension compared with young controls (86, 92). NKG2D is another surface marker of NK cells, which can bind to a variety of protein ligands, including MICA/B (major histocompatibility complex class I chain-related molecules A/B) and ULBP (UL16-binding protein), to activate the killing effect of NK cells (93, 94). The effect of age on the expression of NKG2D in NK cells is controversial. Despite the expression of NKG2D in CD3^-^CD56^dim^ NK cells only significantly increased in neonatal and middle-aged populations (92), other studies have also found that the expression of NKG2D decreased with age (95–97). In addition to CD16 and NKG2D, aging also affects the expression of other receptors on NK cells. For example, the expression of CD57, an inhibitory receptor, increases with age in CD56^dim^ NK cells (86, 92, 97–99). CD57 is thought to reflect the accumulation of highly differentiated, senescent NK cells, which may contribute to age-related immune dysfunction. The expression of KLRG1 in CD3^-^CD56^dim^ and CD3^-^CD56^bright^ NK cells was negatively correlated with age (100). Withal, the expression of inhibitory receptors called killer-cell immunoglobulin-like receptor (KIR) superfamily on NK cells is known to be stable (92, 98) or elevated (in CD56^bright^ NK cells) (85) when comparing adult controls with old subjects. It is important to note that the expression of KIR receptors can be influenced by various factors, the type and content of fatty acids in the plasma of the elderly may be the main influencing factors. Recent studies have found that arachidonic acid (AA) can inhibit the expression of KIR2DL1/S1 and KIR2DL5 in aged NK cells, while DHA and EPA can promote the expression of KIR2DL3 and KIR3DL1 (96). However, the NK cell responses of young adults and healthy elderly recipients in this experiment were similar, and the control experiment and paired Student’s t-test were not performed for statistical analysis of young and old recipients after fatty acid administration, thus it is impossible to determine whether the promotion or inhibition is caused by impaired cell function. Furthermore, aging also affects the functional capabilities of NK cells. NK cells, which are known to eliminate diseased cells through natural killer cell cytotoxicity (NKCC) and secretion of cytokines or chemokines (101, 102), were also found to be affected by aging. As people age, the ability of NK cells to lyse specific cells is significantly attenuated, but no age-related statistical changes have been found in the binding ability of NK cells to target cells, which indicates that the level of NKCC gradually declines, and the post-binding defect of target cells is responsible for the decrease of NK cell killing ability (86). Further, the decrease of perforin release from NK cells is responsible for the reduced NKCC in the elderly population (86). A recent study has used NKCC, rather than therapeutics such as senolytics or senomorphics to eliminate senescent cells (103). The researchers found that 40-50% of senescent cells were killed when the effector-target ratio was 1:1 and the co-culture time was 16 hours (103), which suggests that NKCC could be an effective way to eliminate senescent cells and potentially prevent or treat age-related diseases. In addition to the weakening of NKCC, aging also leads to the decrease of IFN-γ, perforin, and granzyme, yet the increase of TNF-α secreted by NK cells (104, 105), which induces NK cell dysfunction or exhaustion. However, some studies disagree with the above point of view (86, 92). NK cells are the main cells that can exert antibody-dependent cellular cytotoxicity (ADCC). As previously mentioned, research indicates that aging does not have a statistically significant impact on the expression of CD16 (86, 92) or CD16-induced cytotoxicity (106) in NK cells. Therefore, the prevailing belief remains that ADCC, which is a measure of CD16 signal transduction, is not affected by the aging process (106–108). However, aging leads to a shift in the composition of human lymphocytes, with CD57^+^ NK cells becoming the dominant cells (99), which are known to mediate more potent ADCC (109).

In conclusion, the effects of advancing age on NK cell phenotypes vary among different organisms (**Table 1**). In human NK cells, CD56 is an important surface marker. Upon aging, the number of CD56^dim^ NK cells in the blood increases significantly, while the number of CD56^bright^ NK cells remains stable. Due to species differences in the phenotype of NK cells, mouse NK cells are commonly classified into distinct subsets based on the expression of CD27, CD11b, and other markers. The distribution and development of NK cells in most tissues of aged mice have age-related defects, which may be attributed to the reduction of terminal mature CD11b^+^CD27^−^ NK cells (88). Other markers of NK cells, such as CD16 and NKG2D have the effect of activating NK cell killing. CD57 is expressed on senescent NK cells, whereas KLRG1 is notably reduced in NK cell subsets of the elderly. Beyond the impact of aging on NK cells, these cells may in turn have an impact on immunosenescence and healthy aging by clearing senescent cells (110). Granule exocytosis of NK cells plays a crucial role in the elimination of senescent cells (111, 112). For instance, perforin-mediated exocytosis is a primary mechanism by which NK cells clear senescent cells. NK-92 cells have been found to eliminate senescent LX2 cells through the activation of NKG2D and its ligand MICA, ULBP2, as well as robust NKCC and granule exocytosis (112). Overall, understanding the varied effects of aging on NK cell phenotypes and their role in immunosenescence and healthy aging could provide valuable insights into potential therapeutic strategies for age-related conditions.

### 2.4 Aging and mast cells

Mast cells, first discovered by Paul Ehrlich in 1878, are a type of immune cell (113). Based on the tissue distribution of cells, they can be divided into connective tissue mast cells (CTMCs) and mucosal mast cells (MMCs) (114, 115). The origin of mast cells has been controversial, but it is currently believed to vary from the stage of development (116). For example, in humans and mice, mast cells first originate from primitive hematopoiesis in the yolk sac during the embryonic period (117–119), whereas they originate from hematopoietic stem cells in the bone marrow in adults (120). Mast cells play a crucial role as effector cells in the innate immune system. However, the impact of age on their cytobiology is not commonly explored. Therefore, this section aims to review the effects of physiological aging on the quantity, phenotype, and function of mast cells.

Studies on the effect of age on the number of mast cells have found that, as a kind of important effector and regulatory immune cells in the skin (121), the count and frequency of mast cells often increase with age. It was found that the dermis of male fetuses and young men had a low proportion of mast cells, whereas the percentage of mast cells in fibroblast-like cells was increased in the dermis of a 66-year-old man by immunohistochemical staining and spatial morphometry (24). The results of this experiment are similar to those of another experiment on photoprotected skin. In this experiment, mast cells were more numerous in aged vs. young skin by 40%, most of which were located in the papillary dermis, with a lower incidence of degranulation (122). However, some studies have found a significant increase in degranulated mast cells in the skin of aged rats by observing the changes in mast cells in albino rats at different ages (123). To explain this contradiction, we put forth a speculation that the increased coefficient of association between vasoactive intestinal peptide-positive nerve fibers and mast cells in aged skin could be responsible for the lower incidence of degranulation (124, 125). However, it should be noted that the paracrine effect of activated mast cells can trigger adjacent mast cells to undergo degranulation, leading to an increase in the incidence of degranulation (126). Intriguingly, although there are changes in the number of mast cells in the aging papillary dermis, the expressions of several mast cell phenotypes, specifically tryptase and chymase, were not found to be altered (122) (**Table 1**). As individuals age, the number of mast cells increases significantly in various organs, such as epididymis (127), ear skin (127), peritoneal cavity (127), intestine (128, 129), brain (129), heart (130), and kidney (130), not just in the skin. The increase in the number and frequency of mast cells may be due to chronic inflammation during the aging process (24, 128). As the extracellular matrix (ECM) accumulates in aging tissues (131, 132), it triggers the release of proinflammatory and cytotoxic compounds such as chymotrypsin, tryptase, CXCL1, TNF-α, and histamine from ECM-attached mast cells (127). On one hand, ECM may serve as a cell connector (133, 134), contributing to the accumulation of mast cells in aging organs, which may be due to the low apoptosis rate of senescent mast cells resulting in high survival rate (127). On the other hand, the toxic compounds released from mast cells may contribute to a reduction in the number of fibroblasts, which in turn promotes the frequency of mast cells (24).

Age may also affect the phenotype of mast cells. For example, CD45 is present in virtually all bone marrow-derived cells (135), including mast cells, and its expression is increased in the dermis of the elderly (24). Although many studies have examined the impact of age on mast cells, the majority have primarily focused on changes in mast cell numbers. There are comparatively fewer studies that have investigated the effects of age on mast cell surface markers such as CD117, FcϵRI, and T1/ST2 (119). Fibroblast growth factor-2 (FGF-2, or basic fibroblast growth factor, bFGF), intertwined with aging (136) and angiogenesis (137, 138), has been found to affect the function of mast cells. In line with the increased number of mast cells, the expression level of FGF-2 was also upregulated in adult rats compared with young rats, indicating that mast cells may play a role in the aging process by fostering fibrosis (130). Additionally, mast cells also have the ability to trigger lipolysis by releasing histamine (139). It has been observed that the secretory activity of mast cells is heightened and there is a substantial increase in the release of histamine in the skin of aged rats (123). The level of histamine can also be upregulated by stroke in an age-dependent fashion, which was found to be correlated with the significant increase of plasma pro-inflammatory cytokines such as IL-6, granulocyte colony-stimulating factor (G-CSF), TNF-α and IFN-γ in aged stroke mice (129). However, this study failed to identify the source of the pro-inflammatory cytokines, therefore, whether these pro-inflammatory factors are produced by mast cells remains to be further proved by subsequent experiments.

In conclusion, chronic inflammation can result in an increased number and frequency of mast cells in various organs and tissues with age. Firstly, ECM can be used as a local cell docking substrate, helping to reduce the apoptosis rate of mast cells in aging organs. This, in turn, contributes to the accumulation of mast cells. Secondly, cytotoxic products attached to ECM can reduce the number of fibroblasts, which indirectly leads to an increase in the percentage of mast cells. Regarding mast cell degranulation, there are conflicting findings. On the one hand, vasoactive intestinal peptide has been shown to inhibit mast cell degranulation, while on the other hand, activated mast cells can initiate degranulation of nearby mast cells in an autocrine or paracrine manner through the histamine pathway and the tryptase pathway effect. Studies investigating the effects of aging on mast cell phenotype have predominantly focused on CD45, a surface marker of mast cells. The expression of CD45 is found to be higher in the dermis of the elderly, suggesting a potential link between aging and mast cell behavior. Additionally, it’s worth noting that mast cells themselves are also involved in aging and lipolysis pathways, which are induced by age-dependent elevation of FGF-2 and histamine, respectively.

### 2.5 Aging and cGAS-STING signaling

Cyclic guanosine monophosphate (GMP)-adenosine monophosphate (AMP) (cGAMP) synthase (cGAS), which was first reported in 2013 as a cytoplasmic DNA recognition receptor, is also believed to activate innate immune pathways by binding to exogenous pathogenic DNA or cytosolic DNA escaped from the nucleus or mitochondria (140, 141). Stimulator of interferon genes (STING, a.k.a. MITA, MPYS, or ERIS), as a downstream signaling ligand of cGAS (140, 141), was discovered in 2008 in the study of antiviral immunity and found to be critical for the innate immune response induced by intracellular DNA (142–145). Since 2017, when people first began to recognize the significance of the cGAS-STING pathway in cellular senescence (146), the role of this innate immune pathway in the organismal aging has gradually been explored.

Cellular senescence, a fundamental process associated with aging, has emerged as a prominent mechanism in recent years and serves as a hallmark of the aging process (147–149). Yes-associated protein (YAP) and transcriptional co-activator with PDZ-binding motif (TAZ) serve as downstream transcriptional complexes in the protein kinase Hippo signaling pathway, activated in response to robust mechanical signals such as high cytoskeletal forces elicited by stiff ECM (150). When the ECM is flawed and experiences a reduction in mechanical force, the nuclear YAP/TAZ is devoid, consequently inhibiting the expression of downstream target genes such as *Lamin B1* and *actin related protein 2* (*Actr2*). As a result, the integrity of the nuclear membrane is compromised, and the DNA becomes exposed to the cytoplasm. Subsequently, cGAS identifies the exposed DNA and generates the second messenger cGAMP, which activates STING and its subsequent signaling pathways. This activation ultimately leads to the release of senescence-associated secretory phenotype (SASP) and the expression of senescence-associated β-galactosidase (SA-β-gal). Consequently, this cascade of events results in cellular senescence and aging phenotypes (151). *Phosphatase and tensin homolog* (*PTEN*), an extensively researched tumor suppressor gene (152), not only plays a crucial role in diverse signal transduction pathways and cell cycle regulation (153, 154), but also holds significant importance in various physiological activities, including cell adhesion, differentiation, senescence, and apoptosis (155–158). It is worth noting that the inactivation of PTEN lipid phosphatase can upregulate the expression of YAP/TAZ and facilitate the translocation of YAP into the nucleus, thereby inducing cell proliferation and migration (153). However, further studies are required to determine whether this process effectively safeguards the integrity of the nuclear envelope, thereby preventing DNA leakage from the nucleus. Such leakage could potentially activate the cGAS-STING pathway, leading to the promotion of aging. Mitochondrial dysregulation is a condition that induces the activation of the cGAS-STING pathway (159). The loss of PTEN-induced kinase 1 (PINK1) protein, which leads to mitochondrial dysfunction, promotes the activation of the cGAS-STING pathway and consequently results in pathological changes in the kidney, particularly renal tubular aging (160).

Humans and viruses share a co-evolutionary relationship. Endogenous retroviruses (ERVs), also known as long terminal repeat (LTR) retrotransposons (161), are remnants of retroviruses that invaded and integrated into the human genome in ancient times, accounting for 7-8% of the human genome sequence and playing a significant role as genetic memories (162–164). The Pol protein encoded by human endogenous retrovirus-K (HERV-K) performs the reverse transcription of RNA to DNA (165, 166), resulting in the addition of extra DNA to the cytoplasm of human mesenchymal progenitor cells (hMPCs). The DNA sensor cGAS recognizes this DNA and activates the innate immune system, inducing cellular senescence and organismal aging (167). Likewise, cGAS-STING pathway in the brain is also activated with advancing age (168). The inhibition of B-type lamin expression, along with the subsequent resurgence of ERVs, emerged as the primary trigger in the cascade of neuronal senility when human neurons were cultured for prolonged durations. By employing a siRNA gene silencing system that specifically targets the ERV or cGAS pathway, the suppression of aging in human neurons has been successfully achieved (169). However, the study into the aging mechanism focused solely on an *in vitro* model. To fully comprehend the mechanism of ERV-induced cellular aging through the cGAS-STING pathway, it is imperative to conduct future *in vivo* experiments, which will provide further insights and a clearer understanding of the intricate process.

Various neurological diseases including frontotemporal dementia (170), Alzheimer’s disease (171), Parkinson’s disease (172), Huntington’s disease (173), ischemic brain injury (174, 175), amyotrophic lateral sclerosis (176), and ataxia telangiectasia (177) are also germane to aging. These diseases are characterized by an acute or chronic neuroinflammatory response triggered by the cGAS-STING pathway, which significantly contributes to the pathological progression of the conditions (178, 179). Recent studies have not only linked the activation of the cGAS-STING pathway to neurological degeneration (180) and cardiac dysfunction (181) caused by aging but have also shed light on its association with age-related endothelial dysfunction (182). The aging-induced decline in endothelium-dependent vasodilation was significantly inhibited by knocking down cGAS using siRNA or by injecting the cGAS inhibitor RU.521 (182, 183). Consistently, comparable outcomes were observed when using si-STING or the STING inhibitor H-151 (182, 184).

**Figure 2:**
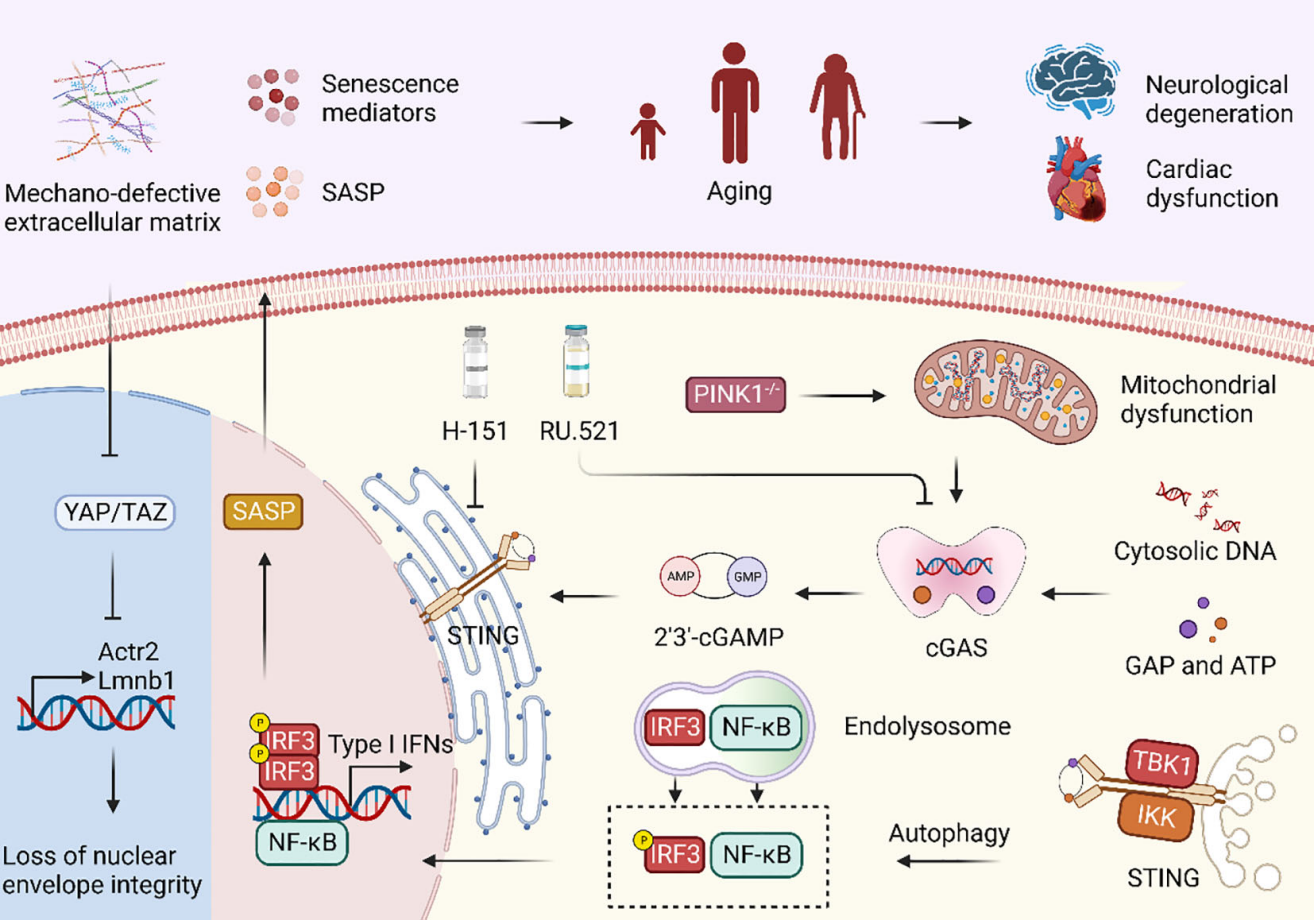
cGAS-STING signaling pathway regulates immune system in aging. Mechano-defective extracellular matrix (ECM) triggers the inactivation of nuclear YAP/TAZ, resulting in diminished expression of ACTR2 and LaminB1, along with the loss of the actin cap and nuclear deformity. Subsequent exposure to cytoplasmic DNA and its binding to cGAS facilitate cGAS-STING activation, ultimately causing cellular senescence and the manifestation of aging phenotypes. Moreover, PINK1 deficiency is correlated with mitochondrial dysfunction, which can also activate the cGAS-STING signaling pathway. Treatment with the STING inhibitor H-151 and the cGAS inhibitor RU.521 both demonstrated a reduction in the expression of senescence signaling mediators and SASP, thereby highlighting their potential as therapeutic interventions.

Overall, the cGAS-STING pathway induces aging primarily indirectly through cellular senescence (**Figure 2**). Under the action of external factors such as extracellular matrix defects, reduced mechanical force, loss of PINK1 protein, and the resurrection of ERV, DNA is released into the cytoplasm to activate cGAS and generate 2’,3’-cGAMP. This molecule binds to STING on the endoplasmic reticulum, resulting in the production of the inflammatory molecule SASP. Ultimately, SASP leads to the death of senescent cells and senescence of adjacent cells, culminating in the aging process. In addition, the cGAS-STING pathway is also involved in the process of considerable aging-related diseases, including neurodegenerative diseases and cardiovascular diseases. Understanding the intricate interplay between the cGAS-STING pathway and these age-associated disorders may pave the way for novel therapeutic interventions and strategies to combat the challenges posed by aging populations.

## 3 Aging and the adaptive immune system

**Figure 3:**
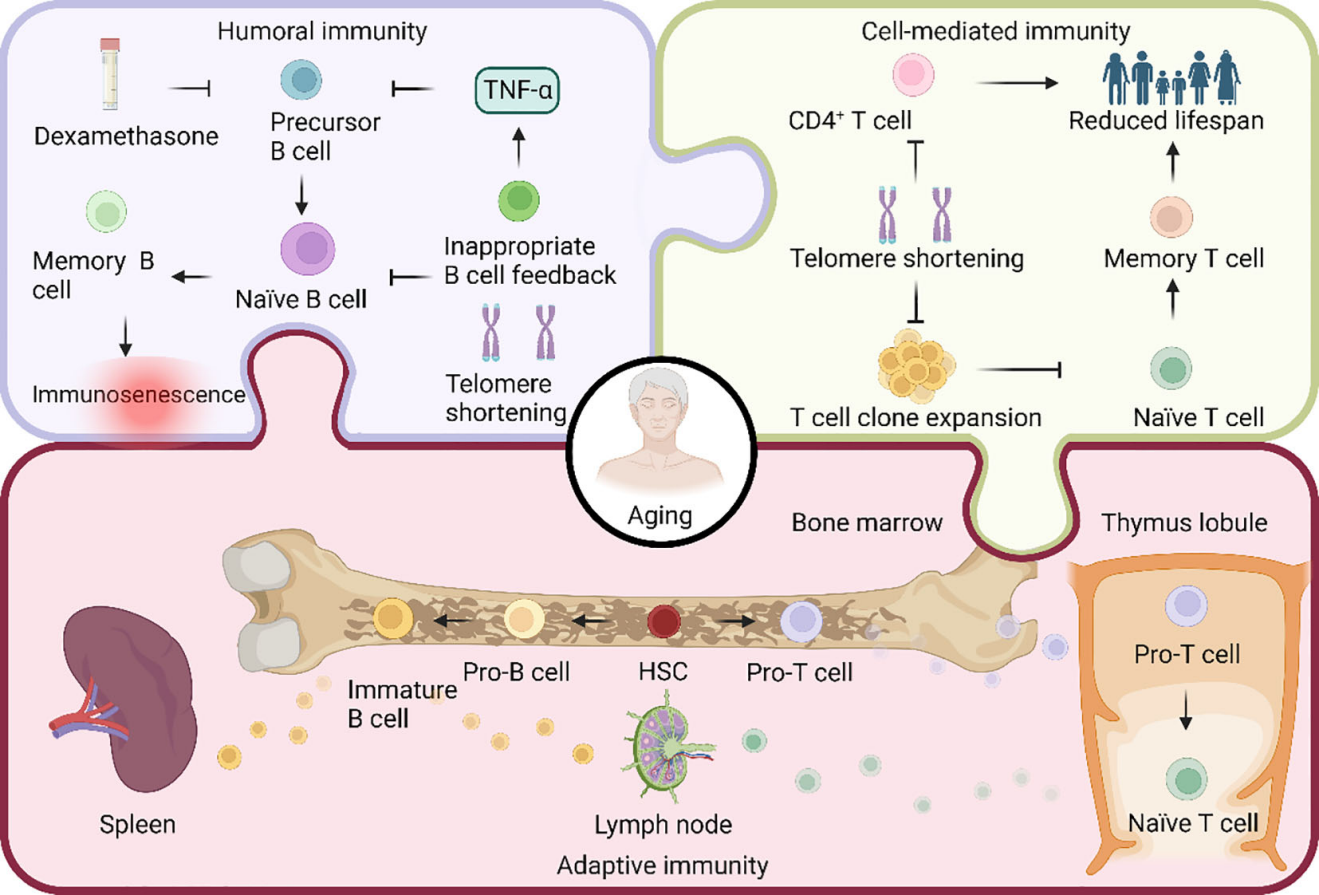
Aging in the regulation of adaptive immunity. Both T cells and B cells are pluripotent stem cells, differentiated from hematopoietic stem cells (HSCs). In the bone marrow, HSCs undergo differentiation, giving rise to progenitor T (pro-T) and pro-B cells. Subsequently, pro-T cells migrate to the thymus, where they undergo somatic recombination to transform into naïve T cells. These naïve T cells then migrate to the lymph nodes. On the other hand, pro-B cells undergo somatic cell recombination and V(D)J recombination, transitioning into immature B cells. These immature B cells migrate to the lymph nodes and spleen to actively participate in the immune response. Adaptive immunity comprises two types: humoral and cell-mediated. B cells mediate humoral immunity, and the reduction in telomere length due to telomerase deficiency during aging hinders B cell production. Additionally, the apoptosis-inducing drug dexamethasone, along with improper feedback from aged age-associated B cells producing TNF-α, inhibits precursor B cell production, ultimately decreasing the overall B cell count. Cell-mediated immunity, on the other hand, is orchestrated by T cells. Age-related telomere shortening results in a significant decline in the expansion ability of T cell clones, leading to a generational reduction in CD4^+^ T cells and, consequently, a decrease in lifespan.

When a pathogen manages to breach the first lines of defense and enters an organism, the adaptive immune system, also known as the specific or acquired immune system, is triggered and begins fighting off the infection (185, 186). Immunosenescence is not only associated with the adaptive immune system, but also with the innate immune system, as already stated above (187–189). These changes during immunosenescence involve a series of age-related modifications in both T cells and B cells (**Figure 3**).

### 3.1 Aging and T lymphocytes

During the natural aging process, the immune system undergoes dysregulation, resulting in the development of various immune-related diseases (190). T cells, which are a crucial component of the adaptive immune system, play a significant role in the process of immunosenescence (16). At the same time, they are also subject to age-related effects.

Thymus serves as the primary location for T cell differentiation and maturation, and plays a crucial role in the process of adaptive immunity (11). It is well-established that in humans, the thymus undergoes development during infancy and gradually involutes and atrophies during adolescence (191, 192). The reason for the large size of the thymus in infancy is possibly as a way to prepare for the establishment of the initial T cell repertoire. The involution of the thymus that occurs with age is primarily characterized by the deterioration of tissue structure, a decrease in thymocytes, and a decline in thymus mass. While the precise causes of thymus involution are not fully investigated, recent research has shed some light on the matter. Malnutrition, resulting from either insufficient or excessive nutrition, is often linked to inflammation, increasing the risk of infections and weakening the immune system (193). In the elderly, thymus, the primary immune organ, undergoes histological and functional decline due to aging and nutritional deficiencies, with disorganization of the corticomedullary boundary being a key feature of its involution (194). Nutrient deficiencies frequently occur in the elderly with age (76), and the histology of the aging thymus changes mainly in the destruction of the corticomedullary boundary, where T cells with different developmental stages are separated into cortical and medullary compartments (194). This separation is crucial for proper immune function, but when it fails, central tolerance is compromised, and autoimmune phenomena may develop (195, 196). Further, since the thymus naturally shrinks in size as we age, dietary restriction doesn’t have a robust impact on its size in older adults. Of note, studies have shown that the rate of thymic involution in mice seems to be slower than that of humans (197). However, the mentioned test on corticomedullary boundary of the thymus in young and old age was constructed using a mouse model, and as of now, there is insufficient evidence to suggest that thymic involution caused by nutritional deficiency will manifest similarly in humans. Some soluble molecules are also thought to be associated with age-related thymic atrophy. By establishing an infant thymic organoid model of thymocyte loss to simulate age-related thymus involution, it was observed that the viability and functional marker CCL21 of thymic epithelial cells was significantly increased along with the reduction of thymocytes (192). Conversely, L-selectin, a biomarker considered to be present in living thymocytes, was found to be significantly reduced in thymic organ cultures *in vitro*, but this change was not seen during aging *in vivo* (192).

In addition to the belief that thymic atrophy leads to the decrease of T cell production and the loss of T cell receptor (TCR) diversity (198), there have been new views on age-related T cell changes recently. Aside from heritability and early-life environment (199), aging is the primary factor that has a significant impact on telomere length (200). Developing a model of telomere length-dependent T-cell clonal expansion capacity with age, individuals with average hematopoietic cell telomere length at twenty years of age maintained maximal T cell clone expansion ability until their sixth decade of life. After this point, this capacity declines exponentially. Additionally, the generation of new naïve T cells and the capacity of naïve T cells to produce memory T cells were also impaired (201). Although naïve T cells possess telomerase capable of reversing telomere length (202), it is clear that this degree of telomere elongation is far from compensating for age-dependent telomere shortening. The telomere length of young mice is longer than that of newborn humans [~50kb (203) vs. ~9.5kb (204)] and telomerase has high activity in somatic cells (205), which may suggest that telomere length-mediated replicative aging is not a concern in the short lifespan of mice. However, the rate of telomere shrinkage is 100-fold faster in mice than in humans (203), which may contribute to their shorter lifespan. Therefore, it is crucial to investigate whether a correlation exists between telomere length and T cell repertoire in aged mice. A study conducted recently used mice deficient in telomerase (mTerc^-/-^) as a model to explore the effects of aging on adaptive immunity (206). The findings showed that in mTerc^-/-^ mice, the number of CD4^+^ T cells in both blood and secondary lymphoid organs (like the spleen and mesenteric lymph nodes) tended to decrease with each successive generation when compared to the control counterpart. The impact of telomerase on naïve CD4^+^ T cells was especially significant. In the third generation of mTerc^-/-^ mice, the frequency of naïve CD4^+^ T cells was significantly reduced in the thymus and spleen and relatively decreased in the blood compared to the control group. In addition, there was no significant difference in the expression of costimulatory molecules CD27 and CD28 (206), which are necessary for antigen activation of immature CD4^+^ T cells through TCR (207, 208), in different generations of mTerc^-/-^ mice. This is different from the up-regulation of co-suppressor molecules CD244 and CD160 in CD8^+^ T cells of the elderly, where high levels of CD244 are also related to the aging of CD8^+^ T cells (209).

Apart from the negative alterations mentioned earlier, aging also causes an elevation in the number of T cells. For example, in aged mice, there was a notable decrease in the output of naïve T cells, while the proportion of memory T cells significantly increased (210). Also, in the subventricular zone of healthy and neurodegenerative elderly people, a substantial rise in the number of CD3^+^ and CD8^+^ T cells was found (211). However, the precise mechanism behind this increase in T cell count remains unclear.

Extensive research has been conducted on the impact of aging on the diminished T-cell repertoire, primarily attributed to thymic involution. Nonetheless, emerging evidence suggests that aging further alters the mechanical characteristics of cells and the internal organization of their diverse components. Various types of cells (212), including T cells (213), experience cellular stiffening as a result of aging. This means that T cells become less deformable with age. Cell deformation is a critical step for T cell activation and migration (214). When T cells become less deformable, their ability to migrate is reduced as well. Recent studies have confirmed that T cells are not exempt from age-related cellular stiffening, as evidenced by increased relative size of nuclei and reduced myosin content, indicating that aging reduces their ability to migrate effectively (213).

Taken altogether, T cells undergo a process of differentiation from naïve to mature cells during development (**Table 2**). T cell recruitment is associated with the generation of new T cells in the thymus. However, as individuals age, the thymus gradually shrinks and becomes involuted due to factors such as malnutrition, resulting in a continuous reduction of naïve T cells. Furthermore, during age-related *in vitro* thymic involution, researchers observe a decrease in L-selectin-labeled viable thymocytes. Surprisingly, despite this decrease in thymocyte content, the thymus actually produces more of the thymocyte chemoattractant CCL21. This suggests that the loss of thymocytes may activate homeostatic mechanisms attempting to counteract the underlying atrophy by enhancing the recruitment of T cell precursors. Unfortunately, these efforts prove unsuccessful in the context of aging. In addition to thymic atrophy and involution, shortened telomere length, decreased telomerase activity, and accumulation of the co-inhibitory molecule CD244 in the elderly may all lead to changes in the number of certain T cell subsets. The function of T cells will also gradually become dysregulated with age, which is specifically reflected in the reduced migration ability caused by T cell stiffening. Nevertheless, it’s worth noting that the effect of aging on the number of T cells is not always negative. The reduction of naïve T cells is often accompanied by an increase in the number of memory T cells and effector T cells. CD3^+^ and CD8^+^ T cells are also significantly increased in the subventricular zone of the elderly. At present, ongoing studies are focusing on artificial thymic organoids (215), presenting a promising new tool for investigating T cell differentiation and reconstruction. Concurrently, it is imperative to gain a deeper understanding of the interactions between T cells and other cell types, their crucial role in maintaining immune balance, and their effectiveness in combating diseases. By delving into these studies, we can advance the prevention and treatment of immune aging, while also devising more effective therapeutic strategies to improve human health.

**Table 2:** Changes in adaptive immune system during aging.

Immune cells	Models	Expression changes	Mechanisms	References
**Naïve T cells (CD62L^High^ CD44^low^)**	Telomerase-deficient mice	Decrease	Telomerase deficiency makes T cells in lymphoid organs more susceptible to apoptosis, impacting the early developmental stages.	(206)
**CD62L^High^ CD44^low^/CD62L^low^ CD44^High^ naïve/memory T cells**	D-galactose-induced aging model mice	Decrease/increase	As the thymus undergoes atrophy with age, the output of naïve T cells decreases, and memory T cells become the dominant cells in the peripheral T cell pool.	(210)
**CD3^+^ and CD8^+^ T cells**	Human aged subventricular zone	Increase	Cytotoxic T cell infiltration is present in the subventricular zone in the elderly.	(211)
**CD4^+^ T cells**	Telomerase-deficient mice	Decrease	The quantity of CD4^+^ T cells is associated with either telomerase deficiency or telomere shortening.	(206)
**Progenitor B cells**	Miz-1^ΔPOZ^ mice	Frequencies decrease, while numbers are stable compared to the control group	The quantity of murine pro-B cells remains consistent throughout the aging process, whereas the aging process notably impacts the maturation into pre-B compartment.	(219)
**Progenitor B cells**	IL-7-mediated mouse pro-B cells	Decrease	In aging, a proportional increase exists in spleen and bone marrow ABCs, acquiring the ability to produce TNF-a and inhibiting pro-B cell growth.	(218)
**Precursor B cells**	IL-7-mediated mouse B cell precursors	Decrease	Coculturing bone marrow cells with splenic age-associated B cells reduces B-cell precursor growth, with inhibition extent depending on ABC quantity.	(218)
**Precursor B cells**	Miz-1^ΔPOZ^ mice	Decrease compared to the control group	The elimination of Miz-1 in B lymphocytes hinders the differentiation process of late precursors starting from the pre-B cell stage onward.	(219)
**Precursor B cells**	BALB/c mice	Decrease	Old age can lead to the loss of pre-B cells, primarily attributed to an elevated rate of apoptosis.	(220)
**Precursor B cells**	C57BL/6 mice	Decrease	The proportion of pro–B cells expressing *rag2* is diminished, and this reduction is associated with a decrease in the quantity of pre–B cells.	(221)
**Precursor B cells**	C57BL/6 and NG-BAC transgenic mice	Decrease	Attrition in the pre-B cell pool is primarily caused by reduced pro-B cell differentiation, resulting from suboptimal pre-BCR signaling and/or decreased synthesis and responsiveness to IL-7.	(223)
**Naïve B cells**	Epstein Barr virus-immortalized human B cell lines	Decrease	Cellular senescence partly leads to reduced B cell function and antigen sensitization capacity with age, ultimately affecting B cell class switching during antibody production.	(224)
**Naïve B cells**	Miz-1^ΔPOZ^ mice	Decrease	Mice lacking Miz-1 exhibit indications of premature aging within the B cell compartment.	(219)
**Naïve B cells**	Human peripheral B cell subsets	Frequency: increase, number: decrease	The absolute number of both naïve and memory B cells is lower in the aged subject compared to the young subjects, the rate of reduction in the absolute number is notably higher in memory B cells than in naïve B cells.	(225)
**Naïve B cells**	Healthy Malawians	Decrease	The thymus undergoes involution with age, leading to reduced production of naïve T cells.	(226)
**Age-associated B cells (CD21/35^-^ CD23^-^ mature B-cells)**	Spleen/bone marrow of aged mice	Increase/Number peaks at 24 month and then declines, frequency increases with age	ABC grows more dominant in the spleen and bone marrow, they might form a subset of B cells that can inappropriately inhibit B lymphopoiesis as individuals age.	(218)
**CD27^+^ B cells**	Human peripheral B cell subsets	Decrease	Memory B cell depletion is a common phenomenon observed in older humans.	(225)
**CD27^dull^ memory B cells/CD27^bright^ memory B cells**	Human peripheral blood	Decrease/increase	The immune systems of elderly individuals may effectively recognize and respond to familiar antigens, yet they exhibit a diminished capacity to react to novel pathogens.	(229)
**CD21^+^ memory B cells**	Human spleen	Increase	Newly egressing memory B cells are recruited into the spleen, systematically organized archive that undergoes homogeneous expansion and remains conserved throughout age.	(230)
**CD19^+^CD27^+^ memory B cells**	Healthy Malawians	Increase	The expression of CD27 on B cells increases with age, and the B cell subsets exhibit age-related changes, with no significant variations based on gender.	(226)
**IgD^high^CD27^neg^ splenic marginal zone B cells**	Human splenic marginal zone	Decrease	The composition of the B cell pool undergoes changes with age and exhibits features indicative of memory B cells.	(238)
**Follicular B cells**	Aged mouse spleen	Frequency: Peak at 12-month-old Number: No significant change	Follicular B cells exhibit heightened CXCR5 expression, yet demonstrate reduced migration in response to CXCL13.	(236)
**Marginal zone B cells**	Aged mouse spleen	Frequency: Peak at 15-month-old Number: No significant change	The impact of aging on the abundance of marginal zone B may vary depending on the background strain.	(236)
**Marginal zone B cells**	Aged female mouse spleen	Frequency: decrease	The decline in the frequency of marginal zone B cells is associated with a concurrent decrease in marginal zone macrophages.	(237)

### 3.2 Aging and B lymphocytes

B lymphocytes derive from progenitor B (pro-B) cells in the bone marrow, mature in the spleen, and then migrate into peripheral body fluids. Once stimulated by antigen, B cells undergo proliferation and differentiation, ultimately producing a large spectrum of plasma cells. These plasma cells produce specific antibodies, which play a critical role in the immune response within body fluids (216, 217).

Both the percentage and absolute number of B cells are known to decline during aging, which may be coupled with improper feedback from age-associated B cells (ABCs) (218) and telomere shortening caused by telomerase deficiency in the elderly population (206). With old age, aged ABCs secrete TNF-α, which hinders the production of precursor B (pre-B) cells (218). A recent study examined the effects of knocking out the Myc-interacting zinc finger protein 1 (*Miz-1*) gene in mice on the number and frequency of different types of immune cells as they age. The results showed that, compared to the control group of mice, there was no significant difference in the frequency of pro-B cells between the two groups as they aged. However, the frequencies of B cells and pre-B cells in the bone marrow were significantly lower in the mice lacking the *Miz-1* gene (219). Administering dexamethasone, an apoptosis-inducing drug, to young adult mice observed a loss of pre-B cells similar to that seen spontaneously in aged mice, suggesting that aging-induced apoptosis may drive the loss of pre-B cells (220). In addition, a decrease in the expression of *rag2* and recombinase activity in pro-B cells during aging may also lead to attrition of pro-B cells during passaging, resulting in a decline in pre-B cells (221). IL-7 plays a crucial role in the transition from pro-B to pre-B cells (222). Therefore, it is possible that the reduction of IL-7 levels during aging may also contribute to changes in the number of pre-B cells (223).

Naïve B cells are developed in the bone marrow from pre-B cells (216, 217), and their numbers also decline with age. This age-dependent reduction may be related to exogenous pathogen infection and deletion of certain genes in addition to pre-B cells. For example, when human B cell lines were infected with Epstein Barr virus, B cells underwent a switch from IgM-producing naïve B cells to IgA- and IgG-producing B cells, this phenomenon is particularly prevalent in B cells from the elderly (224). Additionally, it was found that the depletion of the *Miz-1* in mice led to a significant decrease in the population of naïve B cells (219). However, inconsistent results have sometimes been obtained as to the effect of aging on the percentage of naïve B cells. For example, the proportion of circulating CD27^-^ B cells, regarded as naïve B cells, in the blood was significantly higher in older donors than in their younger counterparts (225), which may be due to the fact that the decline in CD27^+^ B cells is more prominent than that of CD27^-^ B cells with increasing age. On the other hand, research conducted in Malawians discovered that the frequency of naïve B cells was greatest in newborns and then decreased as they age (226).

In the aged immune system, memory B cells play a crucial role and can be distinguished from naïve B cells by differences in phenotype, the responses they exhibit after exposure to activating stimuli, and differential expression of CD27 particularly in humans (227). As a memory B cell subset, ABCs persist in the immune response of the body to external infection (228). In the experiments on mice, the number and proportion of ABCs in the spleen of C57BL/6 mice gradually increased with age, while the count and percentage of ABCs in the bone marrow were variable, with a peak at 24 months old, followed by a gradual decrease (218). Additionally, it has been observed that the frequency and number of peripheral memory B cells with CD27 as a surface marker are higher in young people but lower in older individuals (225). Carsetti’s group has also reported a similar trend (229). As individuals age, the number of memory B cells in the spleen also steadily rises, and these memory B cells of the elderly can make up as much as 60% of all B cells in the spleen (230). Nevertheless, contradictory results were found in the frequency of memory B cells in elderly subjects in Malawi, in line with the previously reviewed naïve B cells, suggesting that the frequency of CD19^+^CD27^+^ memory B cells increases with age (226). The reasons for these contradictory findings could potentially be intertwined with ethnicity (231, 232). In humans, marginal zone B cells are believed to be equivalent to the long-lived IgM memory B cells found in mice (233). Along with follicular B cells, both of which are part of the B2 cell lineage (234, 235). Aging also has an effect on these B cell populations. The frequency of follicular and marginal zone B cells in the spleen increased significantly in 12- or 15-month-old female C57BL/6J mice respectively, but decreased at later ages. For absolute numbers, when comparing 2-month-old and 18-month-old mice, there was a slight upward trend but no significant change (236). However, another experiment in female BALB/c mice showed that the percentage of marginal zone B cells in the spleens of older mice was about 40% lower than that of younger mice (237). This contradictory phenomenon may be related mainly to the different strains of mice used in the experiments. Similarly, this age-dependent reduction was also observed in human IgD^high^CD27^neg^ splenic marginal zone B cells (238).

In total, changes in B cells during aging can be reflected in three aspects: cell number, cell subsets, and secretory function (**Table 2**). As we age, the development of B cells in the bone marrow becomes impaired, leading to reduced production of B cells. Additionally, the aging process also influences the composition of B cell subsets in the body. One notable change is the decrease in the number of pre-B cells during aging, which has been associated with factors such as TNF-α secretion, loss of *Miz-1*, increased apoptosis, decreased *rag2* expression and recombinase activity, and lower levels of IL-7 in ABCs. A significant hallmark of immunosenescence is the decline in naïve B cells and the simultaneous increase in memory B cells (239). In humans, these two cell subtypes are often differentiated by the expression of CD27. The loss of pre-B cells and the susceptibility to pathogen infections are the primary factors contributing to the reduced tolerance of naïve B cells in older individuals. However, the frequency of naïve B cells during human aging sometimes shows variation across experiments, which could be due to the influence of other B cell subtypes counteracting the changes in naïve B cells, as well as differences in ethnicity. On the other hand, memory B cells tend to increase with age and exhibit heightened production of cytokines like IL-1, IL-6, and TNF-α (240), which may contribute to the formation and maintenance of a chronic inflammatory state in the body. Similar to naïve B cells, changes in the number and frequency of memory B cells during aging can also vary slightly between individuals. Understanding the changes in B cells during aging is essential for comprehending age-related alterations in the immune system. Further research is required to shed light on the precise mechanisms that underlie these changes and their impact on overall health and immunity in the elderly population.

## 4 Aging and gut microbiota

Since the proposal of the nine hallmarks of aging in 2013 (147), recent studies have extended our understanding by identifying gut microbiota dysbiosis as a novel hallmark of aging (241). As an integral part of the dynamic organism, throughout our lifetime, the gut microbiota co-evolves with age, drives the maturation of the host’s immune system and thus contribute to host health (**Table 3**).

**Table 3:** Changes in gut microbiota during aging.

Stages		Models	Core microbiome	Expression changes	References
**Initial stage**	First 3 months of early life	Cesarean section delivered infants	Phylum: Firmicutes (dominated from day 5 to week 4 after birth)	Firmicutes: Shows a gradual decrease from day 5 to month 3 after birth.	(330)
		Cesarean section delivered infants	Phylum: Proteobacteria (dominated from week 6 to month 3)	Proteobacteria: Exhibits a consistent upward trend from day 5 to month 3 after birth.	(330)
		Cesarean section delivered infants	Phylum: Bacteroidetes	Bacteroidetes: Exhibits a reduced abundance from day 5 to week 4, followed by a gradual increase from week 6 to month 3.	(330)
		Cesarean section delivered infants	Genus: *Escherichia–Shigella*	*Escherichia–Shigella*: Shows a rising trend from day 5 to month 3.	(330)
	First 3 months of early life	Cesarean section delivered infants	Genus: *Clostridioide*s, and *Streptococcus*	*Clostridioides and Streptococcus*: From day 5 to month 3, there is a decreasing trend in the abundance.	(330)
		Vaginally delivered infants	Phylum: Proteobacteria, and Firmicutes (dominated from day 5 to day 11 after birth) Genus: No obvious variation tendency	Firmicutes: The abundance noticeably declines from day 5 to month 3.	(330)
	40 days, 3 months, and 6 months after birth	Breast-fed infants	Genus: *Bifidobacterium* Family: Enterobacteriaceae	Proportion gradually decreases as time goes on.	(247)
	1-year-old infants	Non-breast-fed infants	Family: Bacteroidaceae	Become dominant in infants aged 1 year that never follow exclusive breastfeeding.	(248)
	3–4 months	Infants at the mean age of 3.3 months	*Ruminococcus* and *Oscillospira*	The ownership of pets leads to an increased abundance of *Ruminococcus* and *Oscillospira* across various birth scenarios.	(331)
	1 week, 1, 3, 6, and 18 months after birth	Prenatal/early-life dog exposure infants	*Fusobacterium*, *Collinsella*, *Ruminococcus*, *Clostridiaceae* and *Lachnospiraceae OTUs*	Living with dogs has higher levels of *Ruminococcus* sp. in their gut compared to those living in households without pets.	(332)
**Transitional stage**	Weaning period	Mouse	Clostridia and Bacteroidia	The weaning period is marked by a relative expansion of Clostridia and Bacteroidia, ultimately shaping the bacterial composition of the adult microbiota over time.	(252, 253)
	Weaning period	>4 months of age infants	Ruminococcaceae and *Faecalibacterium*	Infants who are weaned after the 4th month of age exhibit a rise in levels of Ruminococcaceae and *Faecalibacterium*.	(248)
	Early weaning period	≤4 months of age infants	Veillonellaceae	Early-weaned infants exhibit Veillonellaceae enrichment, though significance faded post multiple-comparison adjustments.	(248)
**Stable stage**		Human	Firmicutes and Bacteroidetes	Dominant bacterial phyla in the intestinal tract during the stable phase.	(258–260)
		Adults	*Blautia* in the Lachnospiraceae family	The gut microbiota of children and adults during the stable phase differs significantly at the family and genus levels.	(258–260)
		Children	*Bacteroides* in the Bacteroidaceae family		(258–260)
**Recession stage**		*Drosophila melanogaster*	Bacilli, γ-Proteobacteria, and α-Proteobacteria	As age advances, the abundance of Bacilli, γ-Proteobacteria, and α-Proteobacteria tends to increase.	(333)
		*Drosophila melanogaster*	*Acetobacter*	The prevalence of *Acetobacter nitrogenifigens* increases in 29-day samples.	(334)
**Rejuvenation stage**		Centenarians from Guangxi Province of China	*Bacteroides*, *Escherichia-Shigella*, *Prevotella*, and *Blautia*	Centenarians show enrichment in Proteobacteria and potentially beneficial Bacteroidetes, along with increased levels of specific bacteria in long-lived individuals.	(335)
		Centenarians and semi-supercentenarians from Emilia Romagna and the surrounding area of Italy	Christensenellaceae	Christensenellaceae shows an increase in both relative abundance and prevalence among centenarians and individuals aged 105 and older.	(270)
**Rejuvenation stage**		Centenarians from Rugao City, Jiangsu Province, China	*Howardella* and *Rikenellaceae RC9*	These genera might play a potential role in preserving youth or reversing the aging process.	(336)
		Centenarians from Guangxi Province or Rugao City, Jiangsu Province, China	*Odoribacter*	The abundance of *Odoribacter splanchnicu* is elevated in centenarians, suggesting a potential role in supporting overall health.	(335, 336)
		Long-lived families from Hechi City, Guangxi Province, China	Rikenellaceae, Porphyromonadaceae, Mogibacteriaceae, Odoribacteraceae, Verrucomicrobiaceae, Christensenellaceae, and Enterobacteriaceae	Increased numbers among long-lived people.	(337)
		Centenarians from Estonia; Hechi City, Guangxi, China; Sardinia, Italy; Bama County, Guangxi, China; and Japan	*Faecalibacterium*	The population with longer lifespans exhibits a significantly lower presence of *Faecalibacterium*.	(269, 337–340)
		Centenarians and semi-supercentenarians from Emilia Romagna and the surrounding area of Italy	Ruminococcaceae, Lachnospiraceae, and Bacteroidaceae	Abundance decreases with age.	(270)
		Centenarians from India, Italy, Japan, and China	Ruminococcaceae	Increase with age.	(341)

Maternal gut microbiota may influence the maturation of fetal immune cells. Pups born to mothers who were transiently colonized with the *Escherichia coli* HA107 strain during pregnancy showed significant increases in innate lymphocytes and F4/80^+^CD11c^+^ mononuclear cells in their gut (242). Thus, the maternal microbiota may play an important role in shaping the offspring’s immune system. Furthermore, feeding pregnant mothers a high-fiber diet resulted in a predominance of Bacteroidetes in their gut microbiota and increased levels of short-chain fatty acids (SCFAs). This intrauterine stimulation may suppress allergic respiratory diseases in offspring, possibly by increasing SCFAs, which induce regulatory T cells (243). Delivery mode strongly influences neonatal early microbial exposure. Moreover, the abnormal colonization of gut microbiota in infants delivered by caesarean section can prolong postnatal immune immaturity and hinder normal immune development, thereby increasing the risk of future immune diseases (244). Breastfeeding, the first and most natural source of nutrition for newborns, profoundly shapes the infant’s gut microbiota and immune system development. Although a baby’s immune system is not fully mature at birth, the complement components in breast milk can partially compensate for this deficiency, helping the baby resist pathogen invasions (245, 246). *Bifidobacterium*, which belongs to Actinobacteria phyla, was the predominant genus in the intestinal tract of breastfed infants (247), while non-breast-fed infants are predominantly colonized by Bacteroidaceae (248).Interestingly, disrupting the gut microbiota early in life seems to increase the risk of autoimmune and inflammatory diseases later on, which suggests that early antibiotic use may have long-lasting consequences (249). The notion that pets offer immune benefits to human health is rooted in the hygiene hypothesis. First proposed by David Strachan in 1989, this hypothesis suggests that an overly hygienic environment increases the risk of allergic diseases (250).

As the babies grow older, their dietary structure gradually changes, from exclusive breast milk and formula feeding to the addition of complementary foods. This transition makes the baby’s gut microbiota undergo a sharp change, known as weaning reaction (251). During this period, the Bacilli class, and in particular the Lactobacillales order, experience a rapid contraction in the gut, while the Clostridia undergo a significant expansion, co-dominate with Bacteroidia, ultimately reaching adult levels (252, 253). In addition, Ruminococcaceae and *Faecalibacterium* also increased significantly (248). The expanding microbiota at weaning triggers a strong immune response, inducing RORγ^+^ regulatory T cells, whose perturbation can lead to increased susceptibility to immune pathology later in life (252). Nonetheless, the effect of early weaning (≤4 months) on the gut microbiota did not differ at the phylum level while may be distinct at the family level (248). Early introduction of complementary foods was associated with a lower relative abundance of *Bifidobacterium* at 3 months of age. However, it is important to note that prematurely reducing the abundance of *Bifidobacterium* by introducing supplementary foods too soon may hinder their interaction with the immune system, potentially leading to higher levels of inflammation (254).

The gut microbiota of adults is relatively stable (255, 256). The initial colonization of the mammalian gut is pivotal for the maturation of the host’s immune system. The innate immune receptor Toll-like receptor 5 (TLR5) functions as a sensor for bacterial flagellin. In murine models, the TLR5-mediated counter-selection of colonizing flagellated bacteria is limited to the neonatal period. Despite this temporal restriction, the process is crucial in determining the composition of gut microbiota, which subsequently influences immune homeostasis and overall health during adulthood (257). Firmicutes and Bacteroidetes were the dominant bacterial phyla in the intestinal tract during the stable phase (258–260), however, there were significant differences in families and genera between children and adults. The largest distinction came from *Blautia* in the Lachnospiraceae (more abundant in the adult cohort) and *Bacteroides* in the Bacteroidaceae family (more abundant in the child cohort), respectively (260).

Although the gut microbiota maintains a relatively stable composition throughout adulthood, it tends to decline as individuals age (261, 262). Changes in the gut microbiota may elevate the expression of pro-inflammatory factors, contributing to chronic age-related inflammation (263). For example, in the absence of adequate SCFA production, the permeability of the intestinal mucosa increases, allowing intestinal bacteria to pass through and enter the bloodstream. Pathogen-associated molecular patterns of invading bacteria, such as LPS, bind to pattern recognition receptors expressed by immune cells and adipocytes and trigger the production of proinflammatory cytokines, leading to chronic inflammation (264, 265).

The gut microbiota can also influence the education and maturation of immune cells, enhancing their function and immunological plasticity (266, 267). For example, the gut microbiota can impact neutrophil production by regulating myelopoiesis in the bone marrow (266). When neutrophils were exposed to bacterial components (LPS), low doses (1 ng/mL) of small extracellular vesicles heightened their proinflammatory sensitivity, leading to elevated TNF-α, IL-6, ROS, and MCP-1 levels, along with enhanced migration and phagocytic activity (267). These findings suggest that small extracellular vesicles can prime neutrophils to respond more robustly to subsequent infections, a phenomenon known as trained immunity. This enhanced response helps defend against potential invading pathogens, thereby maintaining overall health and forming the basis for adaptive immune responses. Conversely, higher doses (28.1 µg/mL) induced a tolerant phenotype characterized by increased IL-10 production and decreased migration and phagocytosis. These effects were linked to changes in TLR2/MyD88 and TLR4/MyD88 signaling, which were associated with the activation of adaptive pathways in neutrophils *in vitro* (267). Microbiota-derived small extracellular vesicles were shown to modulate the function of mouse neutrophils, exhibiting memory-like features (267). With age, neutrophil proinflammatory activities—such as tissue infiltration, phagocytosis, and the formation of neutrophil extracellular traps—increase (266). However, this increase may not always be beneficial, particularly when not properly regulated (20). Changes in the microbiota can influence these neutrophil functions, potentially impacting immune responses in the elderly (266). Microglia are also innate immune cells with adaptive immune memory (268). Aging significantly alters how microglia respond to pathogen challenges by influencing their developmental state and the characteristics of immune memory (268). Neonatal microglia exhibit greater plasticity and can induce trained immunity in response to ultra-low doses of pathogen stimulation, whereas aged microglia are more likely to develop immune tolerance when exposed to high doses (268). This suggests that microglia may become more likely to develop suppressive immune responses with age, which could help reduce excessive tissue damage during repeated systemic inflammation.

Centenarians provide a valuable model for investigating the connection between longevity and gut microbiota. Negative changes in diverse gut microbiota caused by frailty with age lasted until ordinary old age, and was uncoupled from extreme longevity. Long-lived populations typically demonstrate a significant level of α-diversity and species richness (269). Centenarians, as the population with the longest life expectancy in humans, exhibit unique gut microbiota characteristics compared to elderly people. Previous research has indicated that Christensenellaceae could serve as a potential indicator of remarkable longevity in centenarians (270), recent studies have further explored additional microorganisms linked to prolonged lifespan. The original paper suggested minimal changes in the core gut microbiota with age, but certain bacterial taxa within the core microbiota decrease in abundance, particularly Ruminococcaceae, Lachnospiraceae, and Bacteroidaceae (270). Recent studies have shown that centenarians possess a unique composition and activity of immune cell types (271). However, few studies have explored the connection between their distinctive gut microbiota and the immune system. In the future, combining these two lines of research could provide a more comprehensive understanding of the mechanisms behind longevity in centenarians.

**Figure 4:**
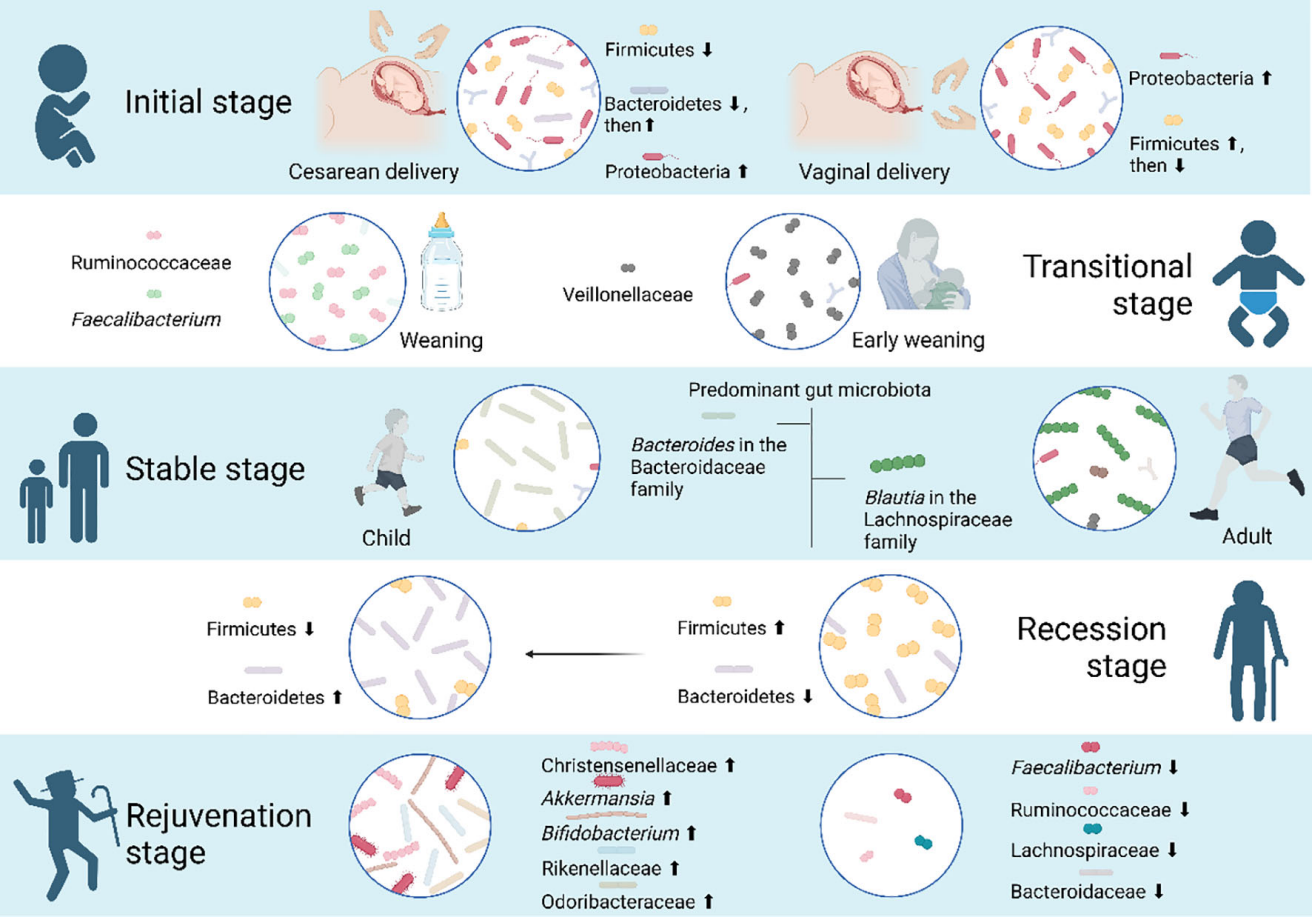
Five stages the gut microbiota goes through during human aging. As individuals age, the composition of the human gut microbiota undergoes distinct phases: an initial phase, a transitional phase, a stable phase, a recession phase, and a rejuvenation phase. In the initial stage, various factors impact the gut microbiota of newborns. Specifically, infants born via cesarean section experience a transition from Firmicutes to Bacteroidetes and Proteobacteria, while those born vaginally have Proteobacteria and Firmicutes as the predominant phyla. During the transitional phase, early weaning has a limited impact on the infant gut microbiota at the phylum level but leads to an enrichment of Veillonellaceae. Notably, Ruminococcaceae and *Faecalibacterium* show significant increases during the weaning stage. By the age of 3, toddlers experience a substantial increase in both the quantity and variety of their gut microbiota, reaching a level of maturity comparable to that of adults, entering a stable stage. However, distinctions persist between the gut microbiota of children and adults. *Blautia* in the Lachnospiraceae family is more abundant in the adult cohort, while *Bacteroides* in the Bacteroidaceae family is more prevalent in the child cohort. As individuals progress into the recession phase of aging, a decrease in the Firmicutes and Bacteroidetes ratio serves as an indicator. Centenarians, in particular, display unique gut microbiota profiles distinct from the average elderly population. Their microbiota undergoes a rejuvenation phase, suggesting that the distinctive microorganisms associated with longevity may play a role in maintaining youth and potentially reversing aging.

In short, as an integral part of the body, the gut microbiota develops alongside the immune system. During the growth and development of the human body from newborns to adults, and then gradually aging, the gut microbiota also undergoes dynamic changes of initial, transitional, stable, and ultimately declining states (**Figure 4**). Centenarians, the poster child for healthy aging, deviate from typical age-related gut microbiota trends, showcasing similarities to youth-associated patterns. Long-lived populations often exhibit heightened α-diversity and species richness. Studying the unique gut microbiota of centenarians offers insights into potential anti-aging strategies, such as fecal microbiota transplantation (FMT).

## 5 Clinical treatment of regulating immunity and aging

Aging, as an irreversible natural process, is closely linked to functional changes in the immune system and alterations in microbiota. These changes not only affect the body’s resistance to pathogens but may also exacerbate pathological conditions associated with aging. Therefore, studying how to regulate immune system function through clinical interventions to delay aging has become a significant focus in current scientific research. In this section, we will explore the latest developments in this field, aiming to provide new ideas and methods for the treatment and prevention of aging-related diseases in the future.

In recent years, a growing consensus among researchers underscores the escalating significance of immune factors in the processes of bodily degeneration and pathological changes, and the onset and progression of numerous diseases are intricately linked to the aging process. Consequently, the exploration of clinical interventions aimed at modulating immunity and retarding aging has emerged as a steadfast focus of ongoing investigation.

A prevalent type of malnutrition among older adults is the lack of essential micronutrients, including vitamins and minerals, which contributes to the gradual weakening of the immune system associated with aging (76). However, the administration of multi-vitamin and mineral supplements (MVM) to the elderly only resulted in a notable increase in the levels of immune-modulating micronutrients, namely vitamin C and zinc, there was no significant alteration observed in the immune function or immune status of older adults (272). Higher levels of micronutrients in the plasma of participants prior to the intervention, the limitations of dihydrorhodamine for measuring ROS (273), the rationality for the choice of specific immune markers (274), and the small sample size of the trial might have constrained the ability to detect significant changes. The beneficial impacts of simple caloric restriction on extending healthspan were initially showcased through rodent studies in 1935 (275), followed by human clinical trials (CALERIE study) in 2015 (276). Recent clinical studies on caloric restriction without malnutrition has also been shown to extend the lifespan of organisms and delay the onset of age-related diseases. For example, two years of caloric intake reduction in humans can rejuvenate the thymus and increase its ability to produce T cells, thereby ameliorating the age-related deterioration of immune function (277). In addition, an 800 kcal/day caloric diet exhibits the potential to postpone immune senescence by shaping the gut microbiota of humanized gnotobiotic mice and regulating immune cell types and proportions (278). Nevertheless, when caloric intake is drastically reduced by as much as 40%, it tends to impede the immune system, thereby increasing susceptibility to more severe infections (279, 280).

Certain senotherapeutic pharmacological agents, such as senolytics that eliminate senescent cells, and senostatics or senomorphics that prevent senescent cells from generating harmful cell-extrinsic effects, also exhibit immunomodulatory properties (281–283). In a groundbreaking *in vitro* experiment conducted in 2015, it was discovered for the first time that the combination of dasatinib and quercetin (D+Q) possesses the remarkable capability to enhance bodily function and extend the span of healthy life by eliminating senescent cells (284). Recent research has expanded upon this perspective. In a preclinical study conducted on the nonhuman primate cynomolgus macaques, the administration of a combination of D+Q (5 mg/kg + 50 mg/kg) for 3 months, the gene expressions of senescence markers *p16^INK4A^* and *p21^CIP1^* exhibited significant reductions. At the 5-month mark, the expression of the apoptosis-related gene *BAX* showed a substantial increase, collectively indicating a notable improvement in the aging status of cynomolgus macaques (285). Aging is often accompanied by a decline in immune capacity. However, D+Q has also been found to possess anti-inflammatory properties. The number of immune cells in the experimental group of animals continued to decrease, allowing the animals to maintain healthier immune characteristics (285). Currently, D+Q combination therapy is being employed in Phase II clinical trials to address mental illnesses linked to accelerated aging (193). Fisetin, another senolytic compound, is abundantly present in various fruits and vegetables (286, 287). Preclinical studies have shown that it has the potential to decrease the number of damaged cells and senescent immune cells in mice, leading to improved health and an extended lifespan in elderly mice (288). These findings suggest that fisetin holds promise for further exploration in human clinical trials. At present, researchers are conducting clinical studies using fisetin as a potential means to target cellular senescence, with the aim of enhancing bone health in elderly individuals (289) and address frailty in the elderly (290). The immune system of the elderly gradually loses its vitality and becomes more susceptible to viruses. Clinical researchers have administered fisetin to elderly COVID-19 patients aged 65 and above to verify whether it can help enhance the immune response in older individuals and consequently reduce the mortality rate associated with the disease (291). In 2016, the senolytic efficacy of ABT-737 and ABT-263 (navitoclax), inhibitors of BCL-2 family proteins (including BCL-2, BCL-W, and BCL-X_L_), was first demonstrated, allowing senescent cells to initiate apoptosis (292–294). Senescent macrophages play a role in the development of lung cancer and tend to accumulate as individuals age (295). However, when young mice with tumorigenic lungs were treated with ABT-737, it led to the ablation of senescent macrophages, thereby enhancing the immunosurveillance process and effectively reducing tumor burden (296). However, due to technical limitations, the experiments fail to provide insights into the exact mechanism through which lung tumors trigger macrophage senescence, nor could it elucidate the reason behind the molecular similarities between senescent macrophages in the lungs of naturally aging mice and those in young mice with lung tumors. Furthermore, and the potential involvement of other macrophage or senescent cell populations in the process of tumorigenesis cannot be excluded. Regrettably, ABT-737’s limited oral bioavailability restricts its potential for therapeutic applications. On the other hand, ABT-263, while offering the advantage of oral bioavailability, presents a significant challenge due to its severe platelet toxicity in clinical settings, rendering it unsuitable for safe human use (297–299).

Age serves as an unalterable risk factor for the inflammation that forms the foundation of age-related conditions like type 2 diabetes mellitus (T2DM) (300, 301). Metformin, as a first-line medication for the prevention and treatment of T2DM in elderly individuals (302), its safety and efficacy have been guaranteed in more than 60 years of clinical use. This extended history of safe use suggests the potential for metformin to also function as an anti-inflammaging medication, promoting healthy aging. Oral metformin, on the one hand, has the ability to repair mitochondria, enabling them to supply energy to cells, restore autophagy function, and counteract the inflammatory response triggered by immune cell Th17. On the other hand, it enhances mitochondrial efficiency and reduces the generation of free radicals by activating the AMP-activated protein kinase (AMPK) pathway, effectively combating the aging process (303). Additionally, metformin has the potential to enhance the growth of beneficial gut bacteria known as *Akkermansia muciniphila*, improve the cognitive function of elderly mice by regulating inflammation-related pathways within the host and lowering the levels of the pro-inflammatory cytokine IL-6 (304). Nevertheless, it remains essential to confirm whether these effects will translate to humans through clinical trials. Ether lipids serve as essential intermediate substances for biguanide drugs, such as phenformin and metformin, which play a pivotal role in promoting life extension, and the biosynthetic state of it also affects dietary restriction, inhibition of mitochondrial electron transport chain, rapamycin, and other aging intervention strategies (305). Rapamycin is known as an immunomodulator (306, 307), and similar to metformin, it is also recognized as a well-established senomorphic. In 2009, the Interventions Testing Program (ITP) certified rapamycin as the first drug to significantly extend the lifespan of mammals, confirming its distinction among dozens of drugs tested for anti-aging research (308). Subsequent *in vivo* animal experiments have conclusively demonstrated that senescent immune cells can induce systemic tissue damage and lead to a shortened lifespan (309). Consequently, senescent immune cells have emerged as a pivotal therapeutic target for extending overall health and longevity. Notably, the administration of rapamycin has been shown to reduce senescence markers in peripheral T cells, boost anti-keyhole limpet hemocyanin (KLH) serum titers, increase white blood cell counts, and effectively reverse the processes associated with systemic aging (309). As individuals age, the deteriorative immune function leads to a diminished response to vaccinations (310). This decline is largely attributed to a reduced capacity of hematopoietic stem cells to generate naïve lymphocytes and an increase in the presence of PD-1 positive T cells. Rapamycin analog RAD001 has been shown to restore hematopoietic stem cell function, reduce the frequency of PD-1-positive CD4 and CD8 T cells, increase the production of naive lymphocytes, enhance influenza antibody titers, improve vaccination response, and extend lifespan (310). Moreover, the decline in immune function that accompanies aging increases the risk of infections, particularly respiratory infections (311). Low doses of RAD001 + BEZ235 (catalytic site mTOR inhibitor) combination synergistically inhibited multiple nodes downstream of TORC1 while avoiding TORC2 inhibition (311). This selective inhibition is significant as TORC2 inhibition is linked to adverse effects like a reduced lifespan in male mice (311). The combination of RAD001 + BEZ235 strengthened IFN-induced innate antiviral immunity, leading to a decrease in infection rates (311). This drug boosts the immune system by upregulating antiviral genes, which may offer broader protection against respiratory infections compared to treatments that target individual viruses. While the anti-aging potential of rapamycin has shown promise in numerous animal experiments (312–316), its transition to clinical applications has encountered significant hurdles. The primary challenge lies in the emergence of toxic side effects when used in humans. For example, rapamycin have been associated with adverse symptoms, including hyperglycemia, hyperlipidemia, nephrotoxicity, impaired wound healing, and immunosuppression (317). However, compared to oral and injectable administration routes, topical rapamycin has, for the first time, clinically demonstrated its ability to delay the aging of human skin tissue (318). This is evident in the increased collagen content observed in the skin of most participants who received rapamycin, as well as the decreased levels of skin cell senescence markers (319). Although rapamycin may affect local immune cell function, unfortunately, no immune cell infiltration was observed in any histological sections from this experiment. In addition, this experiment only observed significant changes in one senescence marker, p16^INK4A^, and future experiments should strive for a more comprehensive assessment of rapamycin’s influence on human tissue aging by examining a broader range of senescence markers.

Research in cell therapy represents a pivotal frontier in the field of medicine. In recent years, significant advancements have been achieved in cell therapy technology, with a focus on immune cells and stem cell regenerative medicine. The primary vehicle for chimeric antigen receptor (CAR) cell therapy is immune cells, originally employed to combat non-solid tumors such as leukemia, B-cell lymphoma, and multiple myeloma clinically (320). In addition to their role in targeting cancer cells, CAR T therapy has firstly found its way into the field of aging biology as a senolytic strategy in 2020 (321–323). In this capacity, animal experiments have shown that CAR T cells can target senescence-specific cell surface markers such as urokinase-type plasminogen activator receptor (uPAR) and natural killer group 2 member D ligands (NKG2DLs), with the potential to eliminate senescent cells, extend survival, and reinstate tissue homeostasis effectively (321, 324). Furthermore, as immune cell reinfusion therapy continues to evolve, T cell receptor-engineered T cell (TCR T) and CAR NK cell therapies are being developed and optimized, with expectations for their potential in anti-aging applications in clinical settings.

In general, current anti-aging interventions can be categorized by their risk levels, with caloric restriction, drugs, and cell therapy spanning from low to high risk. Additionally, other alternative approaches to reverse aging, like gene editing (325) and plasma exchange (326–328), have seen limited adoption primarily because of their cost and ethical concerns stemming from nascent technology. Consequently, there is an imperative to advance these technologies and conduct further clinical trials in the future to establish the safety and efficacy of diverse anti-aging strategies.

## 6 Concluding remarks and prospects

The intricate interplay between immunity and aging presents a captivating realm of study. It encompasses a physiological process entailing intricate mechanisms, encompassing manifold changes in both the phenotype and function of immune cells – both innate and adaptive. The waning vigor and diminishing numbers of these adaptive immune cells can precipitate a decline in the body’s capacity to combat infections and counter aberrant cells, thereby augmenting susceptibility to a spectrum of ailments. Moreover, innate immunocytes assume a pivotal role in regulating inflammatory responses and reparative processes, thereby potential alterations in their functionality could reverberate on the body’s retort to injuries and convalescence. Distinguishing aging from cellular senescence remains a controversial issue. Although cellular senescence is considered a hallmark of the aging process (147–149), some studies suggest that senescent cells *in vitro* and aged cells *in vivo* differ significantly in function and phenotype. For example, microglia cultured *in vitro* exhibit shortened telomeres, reduced cell proliferation, and increased levels of senescence marker proteins, such as p16^INK4a^, p21^CIP1^, and p53. In contrast, microglia acutely isolated from aged brains show only a moderate increase in p16^INK4a^, with no changes in telomere length or cell proliferation (329). This suggests that replicative senescence *in vitro* and aging *in vivo* may involve different mechanisms. In mice, aging *in vivo* appears to be primarily independent of telomeres.

In addition, the aging process is accompanied by dynamic changes in immune-related signaling pathways and gut microbiota. An increasingly robust body of research corroborates the intimate link between the gut and the immune system, underscoring the profound impact of the composition and function of the gut microbiota on immune regulation. With age, these microbial inhabitants of the gut may undergo transformations, thereby potentially exerting a cascading influence on the state and efficacy of the immune system.

Although current research has achieved pivotal strides, a deeper delve is imperative to unveil the intricate molecular underpinnings underpinning the nexus of immunity and aging, along with strategies to potentially stave off the aging process via immune system interventions. This endeavor not only augments our comprehension of the interface between immunity and aging, but also offers novel insights that could potentially shape the trajectory of therapeutic paradigms targeting age-related maladies. Continued in-depth exploration of this field will undoubtedly bring more hope for human health and longevity.
